# Evaluating complex interventions in End of Life Care: the MORECare Statement on good practice generated by a synthesis of transparent expert consultations and systematic reviews

**DOI:** 10.1186/1741-7015-11-111

**Published:** 2013-04-24

**Authors:** Irene J Higginson, Catherine J Evans, Gunn Grande, Nancy Preston, Myfanwy Morgan, Paul McCrone, Penney Lewis, Peter Fayers, Richard Harding, Matthew Hotopf, Scott A Murray, Hamid Benalia, Marjolein Gysels, Morag Farquhar, Chris Todd

**Affiliations:** 1 King’s College London, Cicely Saunders Institute, Department of Palliative Care, Policy and Rehabilitation, Bessmer Road, Denmark Hill, London, SE5 9PJ, UK; 2 School of Nursing, Midwifery & Social Work, University of Manchester, Jean McFarlane Building, Oxford Road, Manchester, M13 9PL, UK; 3 International Observatory on End of Life Care, Faculty of Health and Medicine, Lancaster University, Physics Avenue, Lancaster, LA1 4YT, UK; 4 Department of Primary Care and Public Health Sciences, King’s College London, Capital House, Weston Street, London, SE1 3QD, UK; 5 Department of Health Services and Population Research, King’s College London, Institute of Psychiatry, 16 De Crespingy Park, London, SE5 8AF, UK; 6 King’s College London, Centre of Medical Law and Ethics, Dickson Poon School of Law, Strand, London, WC2R 2LS, UK; 7 Institute of Applied Health Sciences, University of Aberdeen, Cornhill Road, Aberdeen, AB25 2ZD, UK; 8 Department of Cancer Research and Molecular Medicine, Faculty of Medicine, NTNU, Prinsesse Kristinasgt. 1, NO-7006, Trondheim, Norway; 9 Department of Psychological Medicine, King’s College London, Institute of Psychiatry, Weston Education Centre, Cutcombe Rd, London, SE5 9RJ, UK; 10 Primary Palliative Care Research Group, Centre for Population Health Sciences, University of Edinburgh, Medical School, Teviot Place, Edinburgh, EH8 9AG, UK; 11 University of Amsterdam, Centre for Social Science and Global Health, Oudezijds Achterburgwal 185, Amsterdam, 1012 DK, The Netherlands; 12 Primary Care Unit, Department of Public Health & Primary Care, University of Cambridge, Institute of Public Health, Forvie Site, Robinson Way, Cambridge, CB2 OSR, UK

**Keywords:** Palliative care, Terminal care, Research design, Methods, Evaluation studies, Review, Consensus

## Abstract

**Background:**

Despite being a core business of medicine, end of life care (EoLC) is neglected. It is hampered by research that is difficult to conduct with no common standards. We aimed to develop evidence-based guidance on the best methods for the design and conduct of research on EoLC to further knowledge in the field.

**Methods:**

The Methods Of Researching End of life Care (MORECare) project built on the Medical Research Council guidance on the development and evaluation of complex circumstances. We conducted systematic literature reviews, transparent expert consultations (TEC) involving consensus methods of nominal group and online voting, and stakeholder workshops to identify challenges and best practice in EoLC research, including: participation recruitment, ethics, attrition, integration of mixed methods, complex outcomes and economic evaluation. We synthesised all findings to develop a guidance statement on the best methods to research EoLC.

**Results:**

We integrated data from three systematic reviews and five TECs with 133 online responses. We recommend research designs extending beyond randomised trials and encompassing mixed methods. Patients and families value participation in research, and consumer or patient collaboration in developing studies can resolve some ethical concerns. It is ethically desirable to offer patients and families the opportunity to participate in research. Outcome measures should be short, responsive to change and ideally used for both clinical practice and research. Attrition should be anticipated in studies and may affirm inclusion of the relevant population, but careful reporting is necessitated using a new classification. Eventual implementation requires consideration at all stages of the project.

**Conclusions:**

The MORECare statement provides 36 best practice solutions for research evaluating services and treatments in EoLC to improve study quality and set the standard for future research. The statement may be used alongside existing statements and provides a first step in setting common, much needed standards for evaluative research in EoLC. These are relevant to those undertaking research, trainee researchers, research funders, ethical committees and editors.

## Background

There are 57 million deaths each year. Despite being a core business of medicine, end of life care (EoLC) is neglected [1]. Although some people have excellent EoLC, many do not die as they would wish [2]. A major barrier is the lack of quality research; treatments, clinical guidelines and services are limited by a lack of evidence [3,4]. Surveys, qualitative studies and reviews recommend that EoLC research is feasible and ethical [4] but, funding of EoLC research is poor [1,5] and lacks common research guidance. Thus, randomised trials of EoLC treatments and services remain rare, often limited by poor recruitment, high attrition, bias, confounding and small sample sizes [6-8]. There are challenges capturing relevant outcomes in frail patients who may lack capacity, raising ethical reservations [3,6,7,9,10]. Research evaluating EoLC is characterised as too slow, too expensive and frequently not producing useful results [2]. There is a need to improve research methods to evaluate models of service delivery and complex service level interventions in EoLC and identify good research practices to aid future studies. In response, the Methods Of Researching End of Life Care (MORECare) collaboration was established by the UK Medical Research Council (MRC) and National Institutes of Health Research (NIHR) to identify, appraise and synthesise ‘best practice’ methods for research evaluating EoLC. This paper reports the total integrated results from MORECare and the resulting guidance statement.

## Methods

### Design

**Figure 1:**
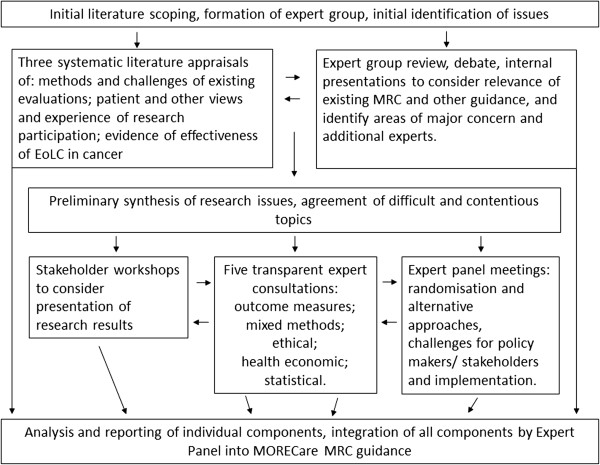
**Diagram showing components of MORECare and how these were integrated.** EoLC, end of life care; MRC, Medical Research Council; MORECare**,** Methods of Researching End of Life Care.

The multiple problems of patients receiving EoLC mean that treatments and interventions are complex, combining symptom relief with physical, emotional, social and spiritual care. We took as a starting point the MRC Guidance for Developing and Evaluating Complex Interventions [11]. We planned a phased study (Figure 1). This involved prioritising areas of uncertainty and difficulties in terms of best research practice in EoLC and developing a statement of best research practice to complement existing tools that aid the conduct and reporting of research, such as the Consolidated Standards of Reporting Trials (CONSORT) for randomised controlled trials (RCTs) [12] or Strengthening the Reporting of Observational Studies (STROBE) for observational studies [13]. We conducted systematic literature reviews, transparent expert consultations (TEC) involving consensus methods of nominal group and online voting, and stakeholder workshops to identify challenges and best practice in EoLC research, including: participation recruitment, ethics, attrition, integration of mixed methods, complex outcomes and economic evaluation. We synthesised all findings to develop the guidance statement.

### Definitions

We defined EoLC as the total or holistic care of a person during the last part of their life, from the point at which a person’s health is in a progressive state of decline, usually in the last year, the last months, weeks or days of life [14].

### Interventions

EoLC includes both generalist and specialist services and is offered across primary, secondary and tertiary care settings [6]. EoLC offers integrated treatments and interventions including specific pharmacological or psychological therapies, education and clinical guidelines (for example, care pathways), patient registers, and direct multi-professional care [6,15].

### Expert panel

We established a panel of experts in trials, quantitative, qualitative and mixed method research, within and outside palliative care, patients/consumers, service providers, clinicians, commissioners, national policy makers and voluntary sector representatives (see Acknowledgements).

### Phase I. Scoping and systematic reviews

We scoped the literature to prioritise areas for systematic literature reviews or consultation (Figure 1). We searched six electronic data bases and reference lists for either systematic reviews of EoLC services, or papers recommending methods in EoLC research, as well as papers recommending methods for the evaluation of complex interventions. Three systematic literature reviews were subsequently conducted, see Table 1[16-18].

**Table 1:** Three systematic reviews conducted and integrated into the final analysis

**Review 1**	To discover the experiences and views of participation in EoLC research of patients, caregivers, professionals and researchers and to identify best practices, we searched seven databases, hand searched three journals and the bibliographies of relevant papers. Inclusion criteria were: original research papers on involvement in EoLC research or its impact on participants. Critical interpretive synthesis (CIS) was used to integrate evidence regarding patient, caregiver, professional and researcher views on, and experiences with, participation in EoLC research, and identify best practices in research participation [17].
**Review 2**	To appraise the state of the evidence of EoLC we conducted a systematic literature review of the evidence of effectiveness of palliative care teams in cancer. We searched six databases augmented by reference lists of earlier reviews. Inclusion criteria were: specialist (that is, with trained and dedicated professionals) palliative care in the home, hospital, or designated inpatient settings for patients with cancer and evaluation of the team. Outcomes were pain, symptoms, quality of life, use of hospital services and anxiety. Studies were excluded if they did not test specialist palliative care services. Meta-synthesis combined the studies according to type of team [16].
**Review 3**	To appraise the methods used and challenges encountered in developing and evaluating palliative and EoLC services we developed the initial scoping into a systematic review specifically addressing this topic. We searched six databases and bibliographies of relevant papers. Inclusion criteria were: systematic reviews on the effectiveness of generalist and/or specialist palliative care (SPC) services for patients with advanced illness and/or their families. Narrative synthesis appraised the methods used against the MRC guidance steps, the main problems encountered and best practice solutions [18].

EoLC, end of life care; MRC, Medical Research Council.

### Phase II. Transparent Expert Consultation (TEC) and Stakeholder Workshops

Five topics were selected for TEC based on results from the scoping (a lack of empirical data) and expert opinion (Figure 1). TEC is a rapid means to agree on recommendations for action, using nominal group techniques to generate recommendations and online ranking to ascertain consensus (see Table 2) [19]. Each TEC followed the same structure. In addition, we considered the conduct and reporting of research in three further workshops - two with patients/consumers and one with clinicians and policy makers. Expert panel meetings were also held every four months and considered randomisation and alternative design approaches, the challenges for policy makers and stakeholders, and the implementation of research findings into practice.

**Table 2:** Transparent Expert Consultation (TEC) process

1.	TEC planning by the MORECare project team, expert panel, and other experts identified in the literature to agree on the focus, scope the literature, and identify topic experts with appropriate multiagency and discipline mix (from health care and clinical research, not only palliative care) for the five workshops. We aimed always to include experts in the methods external to palliative and EoLC, researchers, clinicians, service developers and policy makers in palliative care, patients and consumers.
2..	Specific research questions for each TEC were agreed by the expert panel and included in the invitations sent two to three months in advance
3.	TEC conduct - format: Morning – initial consideration of issues through two or three brief presentations by experts on the subject followed by equal time for discussion. Afternoon – three parallel working groups discussed and generated recommendations on ‘best practice’ to address the issues. Each individual completed a standard form asking them to list specific best practice recommendations to overcome the issues and rank these 1 to 5 (highest to lowest). Members of each group give feedback in turn on recommendations in priority order until the lists were exhausted or time exceeded. Groups discussed recommendations and where possible agreed on the ranking of the importance of the proposals. The afternoon was recorded to ensure that all aspects were captured and individual recommendation sheets and rankings were collated.
4.	Editing of recommendations by the MORECare team to remove duplicates or merge similar proposals and remove any proposals which were strongly generic rather than EoLC specific.
5.	Online consultation on recommendations– inviting all TEC attendees and the MORECare Project Advisory Group, that included the expert panel, to rank each proposal. Participants were asked to rate how much they agreed with each recommendation on a numerical scale from one (strongly disagree) to nine (strongly agree). They were able to make comments on each recommendation and general comments at the end of the consultation.
6.	For each statement we report median agreement to determine the highest ranked items and interquartile (IQ) and total range to determine the degree of consensus. Narrative comments were collated.

EoLC, end of life care; MORECare, methods of researching end of life care.

### Phase III. Synthesis

We planned from the outset to integrate the results from all components to produce overall guidance on ‘best practice’ (Figure 1). We developed the MORECare statement, based on the strongest recommendations from all components of MORECare, as evolving good practice guidance to design and conduct research. This approach is similar to that for tools to support evaluative research (for example, CONSORT, STROBE). Recommendations which went beyond specific study designs were collated for national/international groups.

### Ethics

The research ethics committee of the University of Manchester (reference number 10328) approved the TEC component of MORECare. All TEC participants gave written consent.

## Results

The literature reviews and scoping together identified 15,695 papers, of these 62 were included in the three systematic reviews [16-18]. The results of the scoping and expert panel identified five main areas of contention/uncertainty that required TEC on: ethics [20], statistics (managing missing data, attrition, and response shift), [21] outcome measurement, [22] mixed method research, [23] and health economics [24]. Attendees of the five TECs totalled 140, with 133 responses to the five online consultations (Table 3). The three stakeholder workshops included 19 patients/carers and 12 clinicians. The integrated top ranked recommendations and synthesis with the literature formed: the MORECare statement detailing 36 best practice solutions for research in EoLC to improve study quality and set a standard for research in the future (Table 4); and 13 national/international MORECare recommendations to improve the environment for the development and evaluation of interventions in EoLC (Table 5).

**Table 3:** Transparent Expert Consultation (TEC) considerations and participants

**TEC summit topic**	**Areas considered in TEC workshops**	**Number of attendees**	**Number of online responders**	**Backgrounds of individuals attending and responding**
Ethics[20]	(1) Participation in research; (2) Research ethics committee approval; (3) Informed consent	28	26	ethicists, academics, researchers, members of research ethics committees, clinicians, service providers, commissioners, patients/carers
Outcomes[22]	(1) Outcome measure properties; (2) Optimal time points; (3) Validity of proxy data	31	28	academics, researchers, clinicians, commissioners, experts in outcome measurement
Mixed methods[23]	(1) Phase I and pre-clinical studies; (2) Phase II and III studies and trials; (3) Implementation studies	33	26	journal editors, academics, researchers, clinicians, experts in health services research/mixed methodology, patients/carers
Statistics[21]	(1) Missing data; (2) Attrition; (3) Response shift	20	19	statisticians, researchers, academics, clinicians, patients/carers
Health economics[24]	(1) Cost methods and relevance to EoLC; (2) Outcome assessment; (3) Equity issues.	28	34	health economists, service commissioners, researchers, academics, clinicians, patients/carers
TOTAL		140	133	

EoLC, end of life care.

**Table 4:** MORECare Statement— checklist of components that require consideration when designing and conducting EoLC intervention studies

	**Recommendations**
Introduction/background	1. Present theoretical framework for the intervention and levels of need established
	2. Present objectives appropriate to the level of intervention development
Study design	3. Indicate and justify stage in MRC guidance for development and evaluation of complex interventions, for example, feasibility, preliminary evaluation, efficacy/cost effectiveness and wider effectiveness
	4. Feasibility stages should test both feasibility of the intervention and of methods of evaluation, including outcome measurement
	5. Justify methods, considering appropriate use of existing data sets and secondary analysis as these may produce rapid information
	6. Justify methods of empirical studies considering mixed methods, observational studies and randomised trials
Study team	7. Ensure involvement from: (i) consumers, patients and caregivers; (ii) relevant clinicians; (iii) relevant methodologists to develop study questions, questionnaires and procedures; and (iv) researchers familiar with the challenges in EoLC studies
	8. Ideally, involvement should be well established and continuing, beyond a specific study, with joint meetings or rotations between clinical and research staff
Ethics	9. Note in ethics committee application MORECare recommendations that it is ethically desirable for patients and families in EoLC to be offered involvement in research and MORECare evidence of patient willingness to be approached
	10. Work within legal frameworks on mental capacity, consent and so on, to ensure that those who may benefit from interventions are offered an opportunity to participate if they wish
	11. Collaborate with patients and caregivers in the design of the study, vocabulary used in explaining the study, consent procedures and any ethical aspects
	12. Attend the ethics committee meeting with a caregiver or patient, as a means to help the committee better understand the patient perspective
	13. Ensure proportionality in patient and caregiver information sheets, appropriate to the study design and level of risk, as excessive information in itself can be tiring/distressing for very ill individuals
Participants	14. Adjust eligibility criteria to recruit those patients who may benefit most from intervention, ensuring equipoise
Procedures	15. Minimise burden for existing clinical staff for participation in the study
	16. Clearly distinguish between service received and research activity interviews in study arms when multiple interviews with patients are undertaken in trials, for example, using a graphical system [25]
Outcome measures	17. Choose outcome measures that meet the following criteria:
	• established validity and reliability in relevant population
	• responsive to change over time
	• capture clinically important data
	• easy to administer and interpret (for example, short and with low level of complexity)
	• applicable across care settings to capture change in outcomes by location (for example, patients’ home, hospital, hospice)
	• able to be integrated into clinical care
	• minimise problems of response shift (see below)
	18. Consider including patients’ experience of care, as this is central to many interventions
	19. Select time points of outcome measurement to balance the value of early recording, to reduce attrition, but to allow enough time for the intervention to have had an effect
	20. Consider the potential effect of response shift (that is, a change in a person’s internal conceptualisation or calibration of the aspects measured). Questionnaires that include anchor points or descriptions of each response category may be less problematic in this regard
Missing data and attrition considerations	21. Estimate in advance levels of, and reasons for, attrition and missing data, integrating these into sample size estimates and planned collection of data from proxies
	22. Monitor during the study and report all levels of, and reasons for, attrition and other missing data
	23. Assume missing quantitative data NOT to be at random unless proven otherwise
	24. Test results from different methods of imputation – noting that ‘using only complete cases’ is a form of imputation
	25. Use the MORECARE classification of attrition to describe causes of attrition: that is,
	• ADD – attrition due to death;
	• ADI - attrition due to illness;
	• AaR - attrition at random.
	26. Consider reasons for missing data which are not due to attrition, for example missed questionnaire, or missed data item in questionnaire. Consider these in analysis and the potential imputations
Mixed method studies	27. Mixed methods can be appropriate in all phases of development and evaluation
	28. Ensure appropriate multi-disciplinary skills mix or training of team
	29. Define the theoretical paradigm and method of integrating results and safeguards to ensure rigour at the outset
	30. Plan investigation to avoid undue burden of qualitative and quantitative questionnaires – perhaps dividing data collection or selecting questions and/or sampling appropriately
	31. Take into account any potential therapeutic effect of qualitative interviews where participants can express their feelings, if these are similar to components of the intervention
	32. Ensure that those collecting data are appropriately trained in qualitative data collection
Implementation	33. Consider implementation implications, including workforce and training needs, in all phases of the study
Cost-effectiveness	34. Integrate into preliminary evaluations and test feasibility of methods
	35. Collect data on use of services including health, voluntary, social and informal care, to take societal approach to care costs
	36. Justify appropriate outcome measures to generate cost effectiveness

Note: This checklist should be used alongside other checklists depending upon the specific study design, for example, STROBE, CONSORT. EoLC, end of life care; MORECare, methods of researching end of life care; MRC, Medical Research Council.

**Table 5:** National/international MORECare recommendations to improve the environment for development and evaluation of interventions in EoLC

	**Recommendations**
Ethics	1. Create a Research Ethics Network for Palliative and End of Life care to further and disseminate best practice.
	2. Train those working on ethics and research governance committees in the specific issues and wishes of patients in palliative and EoL care and their families.
	3. Seek to amend the law regarding consent so that advance consent for studies other than clinical trials of medicinal products is legally effective. This would permit research among people who might develop problems with mental capacity later.
Clinician/researcher collaboration	4. Increase collaboration and understanding between clinicians and researchers in EoLC through rotations, joint departments and exchanges
	5. EoLC organisations to create a research-aware culture for practitioners by informing practitioners and patients on admission to a service that the organisation is actively involved in research
	6. Develop specific training for practitioners in palliative and end of life care about research practice, its value and how to recruit
	7. Introduce screening questions about patient/family willingness to be approached for research (as a general principle) in routine initial assessments on entry to palliative care services
Funders	8. Develop collaboration to ensure that funding supports advancement in knowledge, where one study builds from the finding of another and there is progression to multicentre studies, full evaluations and cost effectiveness studies
	9. Assess study proposals against the MORECare statement
National bodies/strategy	10. Develop repositories of routine data and from specific studies which can be used for secondary analysis to quickly answer current questions
	11. Develop collaboration to take forward the MORECare statement
Journal editors/referees	12. In statistical assessment take account of the MORECare statement:
	• that attrition due to death and illness is to be expected and should be planned for when designing EoLC studies. It is not an indication of a poor study unless it is markedly different to that planned, but indicates that a relevant population of patients and families have been included, giving external validity
	• that lack of attrition or missing data is not necessarily a positive finding; it could mean the population studied is less relevant to EoLC
	13. Use MORECare statement to consider good research practice for conducting EoLC studies, alongside established checklists for reporting, for example, STROBE, CONSORT

EoLC, end of life care; CONSORT, Consolidated Standsrds of Reporting Trials; MORECare, Methods of Researching End of Life Care; STROBE, Strengthening the Reporting of Observational Studies.

### Study design recommendations

**Figure 2:**
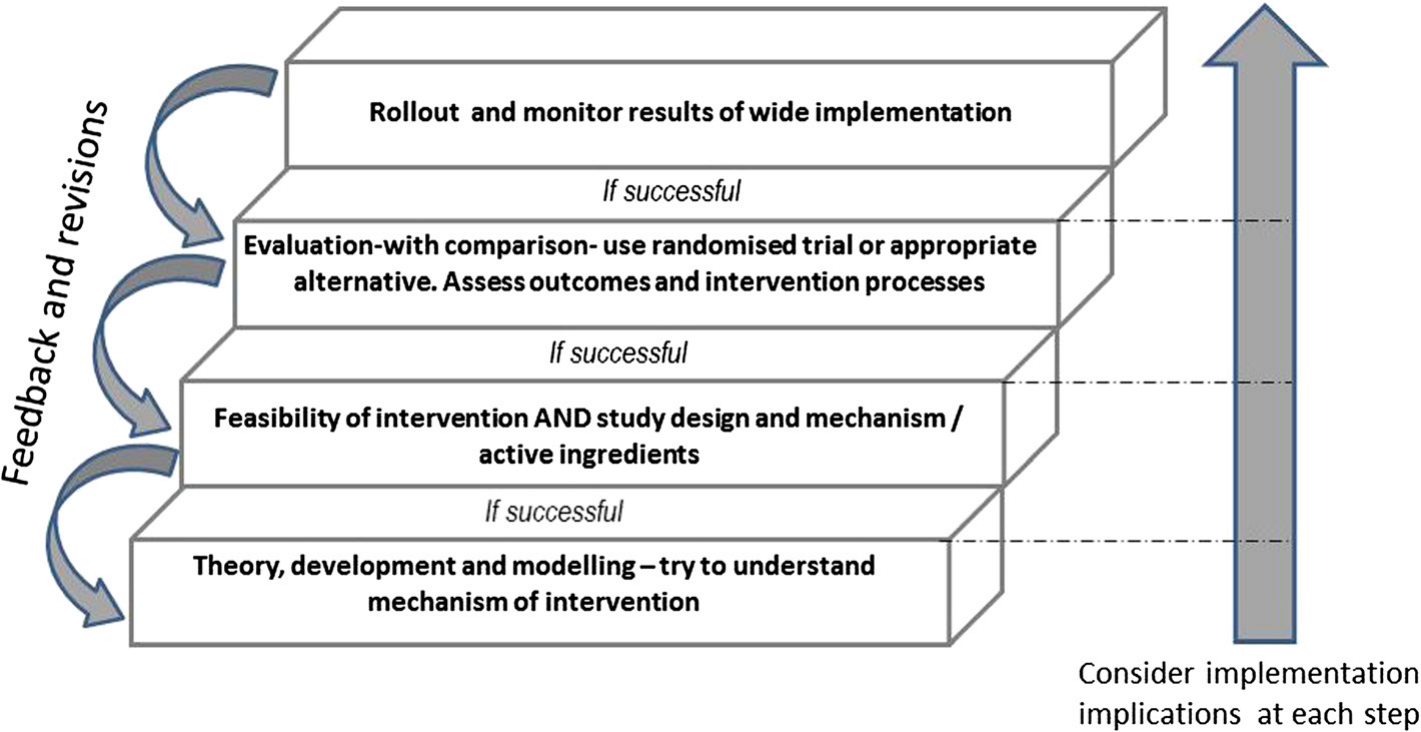
**Key steps in developing and evaluating EoLC interventions.** Although it is possible to begin at any step in the ladder it is important to progress development with successful interventions. EoCL, end of life care.

Three shortcomings for the MRC guidance [11] were identified: (a) moving from feasibility and piloting to implementation without robust evaluation; (b) failing to develop the feasibility of the evaluation methods alongside the feasibility of treatment/intervention; and (c) lack of a theoretical framework underpinning treatment/intervention. In EoLC this has resulted in a lack of pragmatic trials, or, when attempted, trials that fail. There is a need to build simultaneously the intervention and research methods. Understanding the process of the intervention and how it might work is important. Our systematic reviews and expert panel discussions proposed that considerations about implementation be integrated into all phases of evaluation rather than only at the end. This approach ensures that when the intervention is ready to be rolled out, it is feasible with the context and processes of implementation understood, planned for and resourced. The MORECare statement addresses these challenges, see Table 4 and Figure 2.

### Specific aspects in design and execution

#### Patient/caregiver participation

The evidence from the systematic reviews of quantitative and qualitative studies and from the MORECare consultations found that patients (even those close to death) and families were consistently willing to engage in research [17]. Factors reducing such willingness were mainly physical (symptoms, frailty), cognitive impairment or lack of mental capacity. Participating in research was a positive experience for most patients and carers. A minority experienced distress related to the characteristics of the participants, research design (face to face interviews and studies with a clear relevance to care were preferred), or the way it was conducted (very long information sheets, physically struggling to sign a consent form, and poor accommodation of fluctuating symptoms increasing distress). Sometimes the distress, mostly about discussing difficult issues, was acceptable and managed [17].

#### Ethics

Despite the problems of research among individuals who are frail and may sometimes lack cognitive capacity, there was unanimous support across all components of MORECare that it is ethically desirable to offer patients and families the opportunity to be involved in research [20]. Concerns were expressed about an over-protective culture, which sometimes denies patients and families the choice to be involved in research. It can be unethical to assume that patients should not be offered the opportunity purely because they have an advanced disease. Inclusion and exclusion criteria may need to be broadened to allow participation, taking into account any effects on design (Table 4). Methods should take account of expected potential loss of mental capacity. Ethical review and especially governance arrangements were sometimes inappropriate hindrances. Proposals are made for regulatory and legal change to address these (Table 5).

#### Clinician participation

Clinicians are often the first point of contact for EoLC research. There are two aspects: their own willingness to participate and the role they play in aiding the recruitment of patients and/or families. There were mixed reports about health professionals’ attitudes to EoLC research across systematic literature reviews and TECs. Problems can result in poor recruitment due to overt or subconscious control of the recruitment for, or the conduct of, research (sometimes called gatekeeping), which may be influenced by the attitudes towards, and prioritisation of, a research project or research as a whole.

#### Outcome measures and QALYs

Just as treatments in palliative and EoLC are complex, so are outcomes. There is a need to capture changes in symptoms and physical, emotional, social or spiritual needs, at a time when a patient’s condition is deteriorating or death approaches. The measures required will need to be, paradoxically, comprehensive yet brief and sensitive [22]. The MORECare project identified validated outcome measures specifically for palliative care. Beyond traditional psychometric requirements of face, content, and construct validity, the MORECare statement includes other requirements for outcome measures (Table 4). There were, however, strongly opposing views as to whether the commonly used composite measure of outcomes, quality-adjusted life years (QALYs), was appropriate or suitable in EoLC [24]. Debates centred on whether QALYs should be used in palliative care. Participants’ questioned the applicability of QALYs as a measure of outcome for people with life limiting illness and concern that they fail to demonstrate cost-effectiveness. Others argued that QALYs were the most widely used and until alternative measures for palliative care were available the use of QALYs should continue.

#### Statistical approaches; handling attrition and missing data

Attrition and missing data are inevitable in EoLC research. A study which does not have attrition due to death or worsening illness may justifiably be criticised for recruiting the wrong patients [21]. We considered two main approaches to this issue. Firstly, wherever viable, missing data are minimised, using measures which are as short and simple as possible. Where appropriate, proxy ratings from carers or staff may fill gaps which would otherwise exist. Secondly, attrition and missing data are anticipated. Whilst posing a challenge for statistical analysis, they should not be seen as a fault of the study design. Instead, the causes of ‘missingness’ require careful planning for and reporting. We suggest a classification of attrition relevant to EoLC studies to describe causes of attrition (attrition due to death, attrition due to illness, attrition at random). There may not be a single correct statistical analysis applicable for all forms of missing data; however, we suggest an attempt to model the impact of different forms of imputation to test the robustness of study conclusions under different assumptions. These approaches are emergent areas of methodological development and require further debate to identify clear solutions. The statistical analysis plan should include this uncertainty and be prepared and tested while testing the feasibility of the intervention (Figure 2).

## Discussion

This is the first comprehensive research specifically aimed at producing evidence-based guidance for researching treatments and services in EoLC. Our findings propose using randomised trials and other quasi-experimental or observational designs, which may be appropriate when randomisation is not appropriate. Alternative designs would build on traditional RCT methodology and the MRC framework by integrating observational or natural experiment methods, and taking account of implementation aspects, rather than taking a totally different approach. Mixed methods can be employed at all phases of development and evaluation. We found that patients and families value participation in research. Consumer or patient collaboration in developing studies can be valuable in ensuring ethical methods and in addressing the concerns of ethics committees. MORECare also concluded that it is ethically desirable to offer patients and families the opportunity to take part in research and it may be unethical not to offer this opportunity purely on the grounds of progressive disease. Outcome measures should be short, responsive to change and ideally used for both clinical practice and research. More controversially we propose that attrition and missing data should be expected in studies, and does not indicate poor design – indeed, a lack of attrition may mean that the wrong population has been studied. Attrition should be planned for in advance. A new classification of attrition and missing data was developed. Implications for implementation need to be considered at all stages of the project.

MORECare identified the need to involve relevant methodologists, researchers familiar with the challenges in EoLC and consumers/patients/families in studies. The multiple problems of patients mean that interventions are complex, combining symptom relief and physical, emotional, social and spiritual care. The teams required to conduct research in this field, therefore, may also need to be large and complex. Such teams require management, and funding bodies may need to take account of the costs involved.

Our conclusions on the ethical issues raised by EoLC research challenge earlier thinking, especially that randomisation is unethical [17]. There is growing support for the need for research into EoLC to improve practice. The conclusion from the ethics’ TEC was that it can be unethical not to offer research to this group of individuals. Concerns about approaching patients and families who are distressed or very ill are understandable, and this has often led ethics committees or others to raise concerns regarding research in EoLC. However, the vulnerability of patients and families is often simplistically understood. Koffman *et al*. identified five aspects: (i) communicative; (ii) institutional; (iii) deferential; (iv) medical; and (v) social vulnerability, which are relevant in EoLC and other situations, and might provide a broader framework for assessment [26].

The new MORECare classification for attrition: ADD – attrition due to death; ADI attrition due to illness; and AaR attrition at random – is novel, as is our statement that attrition should not be seen as an indication of poor research. Traditionally, guidelines propose that attrition of 5% or lower is inconsequential, whereas 20% or greater is unacceptable because of bias [27]. However, such a guideline fails to distinguish the reasons for attrition. Data which are missed because patients have died is very different to that missed because a patient has withdrawn consent or because they are symptomatic. To impute data such as a quality of life score for a patient who has died seems inappropriate. Whereas imputing data for patients who have moved away or are missed at random would be different. Thus, we designed a classification system for attrition to extend the commonly used classification in clinical trials of missing completely at random (MCAR), missing at random (MAR) and missing not at random (MNAR). We envisage our classification could be used as an adjunct to understand trial data better. Further, while attrition introduces potential for bias, our argument is that a lack of attrition may indicate a different bias, that a less relevant population has been included. In theory, attrition can introduce selection bias in randomized trials. Conversely, a recent secondary analysis from 10 trials evaluating treatment of musculoskeletal disorders, challenged this; the authors found no indication that attrition altered the results in favour of either treatment or control [28]. Work is needed to explore the effect of attrition on bias in EoLC studies, and the best ways to impute data. We believe that our proposed classification will help to clarify reporting and may well be applicable in other populations where attrition is high, for example those who are elderly or frail.

Our findings that outcome measures should be short and easy to use support and further develop conclusions from a large European Network on outcome measurement in palliative and EoLC [29]. We propose further that outcome measurement should be timed to balance the effect of the intervention and loss of data through attrition. Tang and McCorkle proposed an alternative approach, of conducting weekly interviews to ensure adequate data in end of life care studies [30]. While this can be appropriate in some circumstances, it may cause undue interview burden, and we believe the MORECare recommendation of careful timing is more appropriate.

Our proposal that outcome measures used in research should also be valuable in clinical practice is novel, as this is not a usual requirement when assessing outcome measures, although it relates to aspects in the COSMIN (COnsensus-based Standards for the selection of health status Measurement INstruments) checklist of face validity and responsiveness to change [31]. In EoLC there are now many different outcome measures. The European survey identified more than 100 different outcome measures in palliative care research, but 94 of these were used fewer than 10 times [32]. There is a need for standardisation around the few best validated short scales which are widely used, perhaps with core and add on modules [33], so that in the future results from studies may be pooled.

Policy makers, clinicians and patients responding to the MORECare consultation raised the need for research results to be timely to influence service developments. MORECare concluded that robust evaluation data can be found beyond RCTs. This is increasingly raised as an option in research generally [34] and in the most recent formats of the MRC guidance [11]. Secondary analysis of existing data sets, including data collected nationally or in routine clinical practice, and quasi-experimental, epidemiological and qualitative or especially mixed methods can be helpful, especially if the original data is of high quality [34]. Some good examples of secondary analysis of data are available in the USA, where data on hospital activity and costs are routinely available [35].

We attempted to identify the key areas of methodological difficulty in EoLC research; however, we were limited to conducting only five TECs and three systematic reviews, and ideally would have conducted more, especially regarding more specific recommendations on recruitment methods, alternatives to the standard RCT (such as the use of cluster [36] or fast-track trials [37]) and the use of quasi-experimental designs. We see the MORECare statement as a first step, which ideally will be expanded and refined through further testing. Arguably we could have conducted a more traditional Delphi consultation rather than TEC, but the TEC approach allowed a more interactive discussion by allowing novel and sometimes challenging proposals. It did limit our international membership – and only the outcomes summit (which was conducted alongside an international congress) had truly international participation. A particular strength was the involvement of patients and caregivers in all our TECs and throughout the MORECare project, which is uncommon in the development of guidance on good research practice. This involvement resulted in novel proposals, for example, the recommendation for researchers to attend ethics committee meetings with patients, caregivers or consumers came from a patient.

## Conclusions

This research study, which integrated data from three systematic reviews and five TECs, resulted in a statement (the MORECare Statement) of 36 best practice solutions for immediate practice and 13 wider recommendations for national and international consideration. The results show how ethical research is possible, what is required of outcome measures, the need for clinical and academic collaborations and how mixed method research can be reported. Some points in the statements challenge current research practice, for example with new recommendations regarding anticipating, planning for and managing attrition and missing data. Other points require longer term change, for example legal change to permit advanced consent. The MORECare Statement sets clear standards on good research practice in evaluating services and treatments in EoLC. The Statement is relevant to those designing, funding and reviewing studies and should be used alongside existing statements. It provides a first step in setting common, much needed standards for evaluative research in EoLC.

## Abbreviations

ADD: Attrition due to death; ADI: Attrition due to illness; AaR: Attrition at random; CIS: Critical interpretive synthesis; CONSORT: Consolidated Standards of Reporting Trials; COSMIN: COnsensus-based Standards for the selection of health status Measurement Instruments; EoLC: End of life care; MORECare: Methods of Researching End of Life Care; MRC: Medical Research Council; NIHR: National Institutes of Health Research; QALYs: Quality adjusted life years; RCTs: Randomised controlled trials; SPC: Specialist palliative care; STROBE: Strengthening the Reporting of Observational Studies; TEC: Transparent expert consultation.

## Competing interests

The authors declare that they have no competing interests.

## Authors’ contributions

IJH designed the study, led the application for funding, oversaw the MORECare project, contributed to TECs and systematic reviews, led the integration of results, drafted and revised the paper and is the guarantor. CE managed the MORECare project day to day, led one systematic review and one TEC, contributed to protocol development and the integration of the results. GG and CT were co-applicants of the study and contributed to protocol development, TECs, systematic reviews and stakeholder workshops. CT led the Manchester component. NP managed the Manchester component of MORECare, TEC on-line voting and stakeholder workshops. MM, PMcC, RH, SAM, PL, PF and MH were co-applicants of the study, helped to develop the protocol, contributed to TECs and Expert Group presentations. HB, MF and MG led individual TECs or systematic reviews and prepared material to be presented to the Expert Group. All authors commented on and revised the draft paper. All members of the MORECare project contributed to the design and execution of the studies and integration. All authors read and approved the final manuscript.

## Pre-publication history

The pre-publication history for this paper can be accessed here:

http://www.biomedcentral.com/1741-7015/11/111/prepub
